# Editorial: Innovative medical technology based on artificial cells, including its different configurations

**DOI:** 10.3389/fmedt.2023.1306419

**Published:** 2023-11-10

**Authors:** Thomas Ming Swi Chang

**Affiliations:** Artificial Cells & Organs Research Centre, Department of Physiology, Medicine and Biomedical Engineering, Faculty of Medicine and Health Sciences, McGill University, Montreal, QC, Canada

**Keywords:** artificial cells, hemoglobin, antioxidant, carbonic anhydrase, nanobiotherapeutic, regeneration, enzyme therapy, COVID-19

**Editorial on the Research Topic** Innovative medical technology based on artificial cells, including its different configurations

## 1. Introduction

Artificial cell is a highly interdisciplinary area involving medicine, chemistry, bioengineering, biotechnology, biochemistry, and other areas. The highly interdisciplinary approach is such that this Research Topic includes the following participating Frontiers journals: Frontiers in Medical Technology, Frontiers in Bioengineering and Biotechnology, Frontiers in Medicine, Frontiers in Oncology, Frontiers in Molecular Biosciences, and Frontiers in Pediatrics.

**Figure 1:**
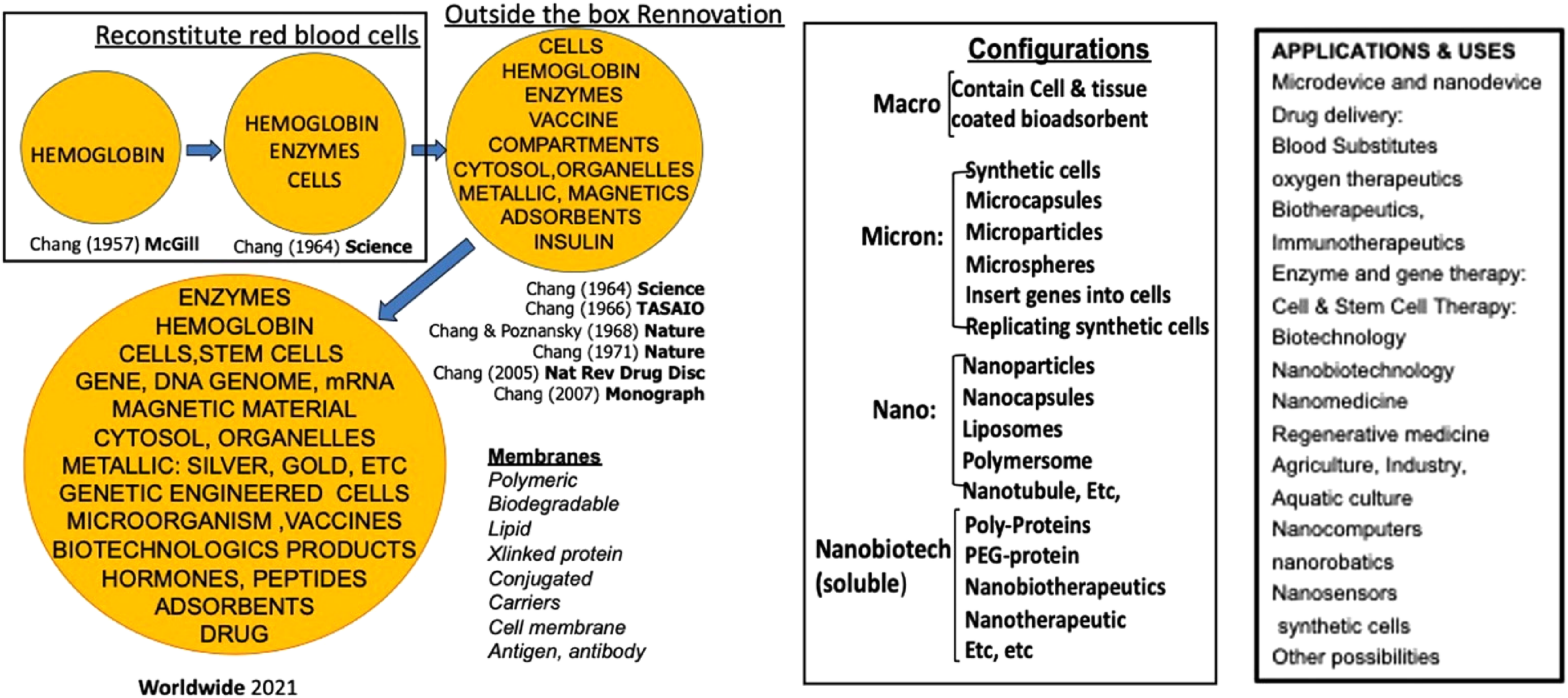
Left: the basic idea of artificial cells evolved into “out of the box variation in contents and membrane materials.” middle: the basic idea of artificial cells extended into different configurations. Right: Examples of uses of artificial cells [Figure from Chang (5) with written copyright permission from the publisher Taylor and Frances].

Red blood cells (rbc) are one of the most important cells since without them, our organs, tissues and cells cannot survive. The first artificial cell prepared is artificial rbc (1, 2). (Figure 1). By going outside the box, we and others are able to extend this to prepare artificial cells of unlimited configurations and contents (2–6). (Figure 1) resulting in large areas of applications (2–6) (Figure 1).

Many of the ideas of artificial cells are being extensively applied and extended by us and by researchers worldwide, resulting in exciting progress and applications (Figure 1). Artificial cell is too large an area to be covered under this journal Research Topic. Artificial red blood cells or blood substitute alone already require a >1,000-page multi-author books (7). Many other areas of artificial cells are in development or routine clinical use. For this Research Topic we shall concentrate on artificial red blood cells as the basis for novel and innovative medical technology and showing how outside the box approaches can lead to innovation application.

## 2. Artificial red blood cell (blood substitutes)

Red blood cells have 3 major functions: 1. Oxygen Carrier 2. Antioxidant functions and 3. transport of carbon dioxide. There was no initial public interest to develop artificial rbc when it was published in 1964 (2). It was the 1980s HIV contamination of donor blood that led to the belated effort to develop a suitable blood substitute.

### 2.1. Oxygen carrier

The urgency was such that researchers concentrated on just an oxygen carrier. In the form of hemoglobin-based oxygen carriers (7). Hemoglobin, a tetramer, is an excellent oxygen carrier. However, in the body it is converted into toxic dimers. Chang used diacid (2) or glutaraldehyde (8) to crosslink hemoglobin into polyhemoglobin (PolyHb) and prevent its breakdown into toxic dimers. This has been developed and tested in clinical trials. A glutaraldehyde crosslinked bovine polyhemoglobin has been approved for routine clinical use for surgical procedures in South Africa and Russia to avoid the use of HIV contaminated donor blood (9). Thus, the original aim has been reached as described in Jahr's article on *Blood substitutes: Basic science, translational studies and clinical trials*.

Polyhemoglobins do not have blood group. Moore et al. reported their clinical trials with glutaraldehyde crosslinked human PolyHb. They showed that this could be given right In the ambulance for patients with hemorrhagic shock without the need for cross matching. This was more effective than the saline control group. They reported a very slight increase in non-fatal myocardial ischemia (10). This could be due to a number of reasons as discussed by Alayash in their article *Oxidation reactions of cellular and acellular hemoglobins: Implications for human health* and Williams et al. in their article *Renal glomerular and tubular responses to glutaraldehyde- polymerized human hemoglobin*.

### 2.2. Oxygen carrier with antioxidant functions

For those conditions with ischemia-reperfusion, one would need an oxygen carrier with antioxidant properties. Thus, our approach of polyhemoglobin-catalase-superoxide dismutase (PolyHb-CAT-SOD) prevents cerebral edema in hemorrhagic shock with cerebral ischemia (11, 12). Another oxygen carrier with antioxidant showed similar results. This is reported by Jun Wang et al.'s group in *Polynitroxylated PEGylated hemoglobin protects pig brain neocortical gray and white matter after traumatic brain injury and hemorrhagic shock.*

### 2.3. Oxygen carrier with antioxidant property and CO2 transport

Another possible problem is an increase in intracellular pCO2 in severe hemorrhagic shock (13, 14). We therefore added an enhanced level of carbonic anhydrase (CA) to prepare a PolyHb-CAT-SOD-CA. The result is an oxygen carrier with enhanced Carbonic Anhydrase for CO2 transport and enhanced Catalase and Superoxide Dismutase for antioxidant functions (15). This is reported by Bian and Chang in this Research Topic. Hoq and Chang used more stable chemical CAT, SOD and CA instead of biological enzymes for certain uses as reported in the Research Topic article *Preliminary feasibility study using a solution of synthetic enzymes to replace the natural enzymes in polyhemoglobin-catalase-superoxide dismutase-carbonic anhydrase: effect on warm ischemic hepatocyte cell culture*.

### 2.4. Other approaches

Many other approaches are being explored (7). This Research Topic shows two examples. One is Kettisen et al.'s group in *Structural and oxidative investigation of a recombinant high-yielding fetal hemoglobin mutant.* Another is Sakai et al.'s group in *Research of storable and ready-to-use artificial red blood cells (hemoglobin vesicles) for emergency medicine and other clinical applications*. Biodegradable polymeric membrane nano-rbc is also possible (16).

### 2.5. Preservation of pretransplant cells and organs: regenerative medicine

A very promising and exciting area is the use of blood substitute in the preservation of cells and organs for transplantation. Zal's murine HEMARINA has been approved in the EU for the pretransplant preservation of human kidneys (17).

Active research continues in this exciting application. A number of years ago we show that oxygen carrier with antioxidants is effective for the preservation of rat small intestine and hepatocytes (18). Andrijevic et al. reported their detailed study in Nature (2022) (19). In this Research Topic, Li et al. describe their work in *Application of polymerized porcine hemoglobin in the ex vivo normothermic machine perfusion of rat livers* alongside Shen et al. in *The role of normothermic machine perfusion (NMP) in the preservation of ex-vivo liver before transplantation: A review*.

## 3. Other areas

Artificial rbc or blood substitutes is but a very small area of artificial cells. Much larger areas of uses have been reviewed elsewhere (Figure 1) (4, 5). A very brief summary of some of these follows.

1. Hemoperfusion: Artificial cells containing bioactive material for hemoperfusion (20–22). One example is the. routine clinical use of artificial cells containing adsorbent for the removal of waste metaboletes and drug poisoning (21,22) and in cytokine storm of severe COVID-19 (23). It also has other uses like the treatment for immunological disease as reported in this Research Topic in *The development of immunosorbents for the treatment of systemic lupus erythematosus via hemoperfusion* by Yu and Ou.
2. Delivery system: Artificial cells in different configurations have used successfully as drug carriers (4, 5, 24, 25). A recent report is Dhasmana et al.’s *Fabrication and evaluation of herbal beads to slow cell ageing*.
3. Artificial Cells in the fight against COVID: The potential of artificial cells as carrier for vaccine has been proposed many years ago (24, 26, 27). Artificial cells prevent mRNA from inactivation by body enzymes and allow it to carry out its function as COVID-19 vaccine (26, 28). Artificial cells based hemoperfusion has also been used to lower the elevated toxic level of cytokines in patients with severe COVID-19 (23, 26).
4. Hereditary enzyme deficiency: We first show that catalase artificial cells can replace the deficient enzyme in hereditary catalase deficient mice (29). Use in patients only became possible when stable simple enzymes became available for artificial cells. This allowed us to treat a patient with Lesch Nyhan disease using artificial cells contain xanthine oxidase (30, 31). We also use artificial cells containing Phenylalanine ammonia lyase (PAL) to treat rats with Phenylketonuria (32). A company collaborated with us to prepared less expensive recombinant PAL which was used by another company to prepare PEG-PAL that has been approved by FDA for adult PKU patients (33).
5. Cancer therapy: We first reported the use of artificial cells containing asparaginase for the suppression of lymphosarcoma in mice (35). This is now being used in patients in the form of PEG-asparaginase. We are using artificial cell containing polyHb-tyrosinase in mice with melanoma (37, 38) Soltys et al.’s group reports in this Research topic in *Inhibition of metastatic brain cancer in Sonic Hedgehog medulloblastoma using caged nitric oxide albumin nanoparticles*.
6. Other areas: Other areas of uses include cell/stem cell therapy and regenerative medicine (39–43), encapsulated microbe (44–46) industry, agriculture, aquatic culture (4, 5, 49), synthetic cells (47), nanomedicine, biotherapeutics, gene therapy, nano-robotics and others (Figure 1) (4, 5, 49).

## Summary

Artificial cells can be prepared with extensive variations in terms of their contents, membranes, dimensions, and configurations. This allows for an array of promising and innovative medical applications. In this Research Topic, we will start with a detailed discussion of the current status and future prospects of artificial red blood cells. Notable developments in this area include hemoglobin-based oxygen carriers, which have been approved for use in surgical patients by two countries. Additionally, researchers are using natural and synthetic enzymes to form oxygen carriers with antioxidant properties or those with CO2 transport and antioxidant properties. Other approaches include bioengineered hemoglobin and hemoglobin vesicles. Another active area of research centers on the preservation of tissues and cells, with Hemarina having received approval in the EU for the pretransplant preservation of human kidneys. Brief discussion of other notable innovative applications of artificial cells encompasses hemoperfusion, delivery systems, COVID-19 vaccines, cancer therapy, and hereditary enzyme defects. The possibilities extend beyond these, as listed in Figure 1, encompassing nanomedicine, biotherapeutics, gene therapy, regenerative medicine, cell and stem cell therapies, and nanorobotics.
